# Supplementary material for: The In Vitro Efficacy of Activated Charcoal in Fecal Ceftriaxone Adsorption among Patients Who Received Intravenous Ceftriaxone
Source: Antibiotics (Basel). 2023 Jan 9;12(1):127. doi: 10.3390/antibiotics12010127 (PMC9854876; doi:10.3390/antibiotics12010127)
Supplement: Supplementary file 1 [file antibiotics-12-00127-s001.zip › antibiotics-2097988-supplementary.pdf]

Supplementary Materials

Mixing protocol planned to mimic the adsorption of ceftriaxone in the human cecum

Our in vitro mixing protocol was set to resemble cecum conditions (Table S1).

Table S1. Comparison of conditions between the cecum environment and mixing protocol in the study.

Conditions	Cecum	Mixing protocol
Temperature (degrees Celsius)	37	37 (incubator)
Light	No	Opaque incubator
Oxygen	Low level	Tightly closed tube lid
pH	Mildly acidic to neutral	Diluted in normal saline (mildly acidic to neutral)
Concentration of contents (xenobiotics, food particles, etc.)	Roughly 0.1x stool	1 to 10 dilution of stool
Concentration of activated charcoal	No data	Activated charcoal per day dose/fecal amount per day (3, 15, 50g/100g)
Movement	Peristalsis	Shaking

It should be noted that the real conditions in the large intestine are dynamic, full of live micro-organisms and mucosal epithelial cells. The concentration of both xenobiotics and activated charcoal could be changed throughout the whole length of the large intestine. Our mixing protocol was static, with unchanged conditions and mostly dead micro-organisms from the heat inactivation. Therefore, the efficacy of xenobiotic adsorption by activated charcoal might be quite different in vivo.

Adsorption efficacy testing among three brands of activated charcoal (AC), commercially available in Thailand

We tested three different brands of marketed conventional AC: brand A, B, and C. We chose only one sample, X_3, to test in this step to conserve budgetary resources, and it was reasonable to answer the question in this step. We found that brand A had higher % adsorption of fecal ceftriaxone than brand B at the same dose of AC 30 mg/g of X_3 feces at 3,000× dilution, while AC 150 mg/g (of feces) of brand C showed significantly higher % adsorption of fecal ceftriaxone than brand A at 300× dilution. We decided to select brand C, which had the highest in vitro efficacy in fecal ceftriaxone adsorption as the best representative AC for the final step in this study (Table S2).

Table S2. Fecal ceftriaxone concentration comparison between 'without' and 'with' AC of different brands and different doses.

**Samples for ceftriaxone level measurement**	**no AC**	**+ AC Brand A**		**+ AC Brand B**		**+ AC Brand C**	
		**30 mg/g**	**150 mg/g**	**30 mg/g**	**150 mg/g**	**30 mg/g**	**150 mg/g**
X_3 (3,000× dilution)	3.5 ng/ml	1.8 ng/ml (48.57% adsorption)	ND	3 ng/ml (14.29% adsorption)	ND	ND	ND
X_3 (300× dilution)	> 40.5 ng/ml	ND	> 40.5 ng/ml (NA)	ND	ND	ND	13 ng/ml (> 67.90% adsorption)
X_{Null} (3,000× dilution)	< 0.5 ng/ml	< 0.5 ng/ml	< 0.5 ng/ml	< 0.5 ng/ml	ND	ND	< 0.5 ng/ml
Negative control (NSS)	< 0.5 ng/ml	< 0.5 ng/ml	ND	< 0.5 ng/ml	ND	< 0.5 ng/ml	ND
Positive control (Ceftriaxone 30 ng/ml)	30 ng/ml	< 0.5 ng/ml (>98.33% adsorption)		< 0.5 ng/ml (>98.33% adsorption)		< 0.5 ng/ml (>98.33% adsorption)	

ND = not done

NA = not applicable

Brand A – pure powder of activated carbon, manufactured by S. Tong Chemicals Co., Ltd., Nonthaburi, Thailand

Brand B – GREATER CA-R-BON®, manufactured by Greater Pharma Co., Ltd., Nakhon Pathom, Thailand

Brand C – DELTACARBON (Medicinal Charcoal)™, manufactured by SPS Medical Co.,Ltd., Bangkok, Thailand

Optimal dilution test

Very high fecal ceftriaxone concentrations, not only of X_x samples but also X_{null} fecal medium, could disturb the chemical reactions of the ELISA assay. Thus, dilutional titration for each sample was necessary. Thirty-times (30×) dilutional factor was found to be the lowest acceptable dilution for which ELISA results could be interpreted. We tested X_1-X_5 samples first and found that final dilutions of 30× to 300× for X_1 and X_2, while 300× to 3,000× were suitable for $X_{3\text{-}5}$ (Table S3.).

We chose 100× final dilution for X_1 and X_2, because 300× dilution was too diluted to detect a difference in the effect of AC in fecal ceftriaxone adsorption. Though X_1 and X_2 had very low fecal ceftriaxone concentrations, they should still be diluted more than 30×, since the presence of high levels of fecal material alone, as in X_{null} (no ceftriaxone) at 30× dilution, could interfere with the ELISA result.

Thus, 2,000× dilution was designated to be the final dilution for $X_{3\text{-}5}$, because 3,000x dilution was also too diluted and 300× dilution was very close to the upper limit of the test detection. Hence, we also use 2,000× dilution for $X_{6\text{-}8}$.

Table S3. Up-titrated dilution of fecal medium for $X_{1\text{-}5}$ and X_{null} fecal medium to find out the final dilution that was suitable for the detection limit of ceftriaxone level by ELISA.

Samples	30× dilution	300× dilution	3,000× dilution
X_1	15 ng/ml	2.5 ng/ml	0.8 ng/ml
X_2	3 ng/ml	1.5 ng/ml	< 0.5 ng/ml
X_3	> 40.5 ng/ml	> 40.5 ng/ml	3.5 ng/ml
X_4	> 40.5 ng/ml	> 40.5 ng/ml	9 ng/ml
X_5	> 40.5 ng/ml	40 ng/ml	4 ng/ml
X_{Null}	0.5 ng/ml	< 0.5 ng/ml	< 0.5 ng/ml
Negative control (NSS): ceftriaxone 0 ng/ml<br>Positive control: ceftriaxone 30 ng/ml			
